# GroEL Chaperonin-Based Assay for Early Diagnosis of Scrub Typhus

**DOI:** 10.3390/diagnostics12010136

**Published:** 2022-01-06

**Authors:** Nitaya Indrawattana, Pisinee Aiumurai, Nawannaporn Sae-lim, Watee Seesuay, Onrapak Reamtong, Manas Chongsa-nguan, Wanpen Chaicumpa, Nitat Sookrung

**Affiliations:** 1 Department of Microbiology and Immunology, Faculty of Tropical Medicine, Mahidol University, Bangkok 10400, Thailand; nitaya.ind@mahidol.ac.th (N.I.); mchongsanguan@yahoo.com (M.C.-n.); 2 Center of Research Excellence on Therapeutic Proteins and Antibody Engineering, Department of Parasitolgy, Faculty of Medicine Siriraj Hospital, Mahidol University, Bangkok 10700, Thailand; pisinee.aiu@mahidol.ac.th (P.A.); nawannaporn.sae@mahidol.edu (N.S.-l.); watee.see@gmail.com (W.S.); wanpen.cha@mahidol.ac.th (W.C.); 3 Department of Molecular Tropical Medicine and Genetics, Faculty of Tropical Medicine, Mahidol University, Bangkok 10400, Thailand; onrapak.rea@mahidol.ac.th; 4 Faculty of Public Health and Environment, Pathumthani University, Pathum Thani 12000, Thailand; 5 Biomedical Research Incubator Unit, Department of Research, Faculty of Medicine Siriraj Hospital, Mahidol University, Bangkok 10700, Thailand

**Keywords:** antigen detection test kit, scrub typhus, GroEL chaperonin, *Orientia tsutsugamushi*, hybridoma, immunochromatography (ICT)

## Abstract

A point-of-care diagnostic for early and rapid diagnosis of scrub typhus caused by *Orientia tsutsugamushi* is required for prompt and proper treatment of patients presenting with undifferentiated febrile illnesses. In this study, an immunochromatographic antigen detection test kit (ICT AgTK) that targets the highly conserved *O. tsutsugamushi* 60 kDa GroEL chaperonin (heat shock protein 60) was developed. *E. coli*-derived recombinant GroEL expressed from DNA coding for the consensus sequence of 32 GroEL gene sequences extracted from the GenBank database was used to immunize rabbits and mice. Rabbit polyclonal antibodies (pAb) were used for preparing a gold-pAb conjugate, and the rGroEL-specific mouse monoclonal antibody was used as the antigen detection reagent at the ICT test line. In-house validation revealed that the ICT AgTK gave 85, 100 and 95% diagnostic sensitivity, specificity and accuracy, respectively, compared to the combined clinical features and standard IFA when tested on 40 frozen serum samples. The test kits correctly identified 10 scrub typhus samples out of 15 fresh plasma/buffy coat samples of patients with febrile illnesses. For independent laboratory validation, the ICT AgTK was sent to one provincial hospital. The ICT AgTK utilized by the hospital medical technologist correctly identified six scrub typhus samples out of 20 serum samples of patients with fever, as confirmed by specific IgM/IgG detection by IFA. The ICT AgTK is easy to perform with rapid turn-around time. It has the potential to be used as an important tool for on-site and early scrub typhus diagnosis by allowing testing of freshly collected samples (serum, plasma or buffy coat), especially in resource-limited healthcare settings.

## 1. Introduction

Scrub typhus is a febrile illness caused by an obligate intracellular and highly pleomorphic Gram-negative bacterium of the Rickettsiales order, Rickettsiaceae family, named *Orientia tsutsugamushi*. The disease predominates in rural areas of the Asia–Pacific region where there are heavy scrub vegetation and forest [1,2,3]. *Orientia tsutsugamushi* consists of six prototypic strains, including Gillian, Karp, Kato, Shimokoshi, Kawasaki and Kuroki [4,5]. Humans are infected through bites by infected larvae (chiggers) of trombiculid mites that live on small mammals such as field mice and rats [6]. The saliva of the chiggers contains an enzyme that can damage human skin, causing a typical necrotic lesion at the wound called an eschar [7]. The incubation period of scrub typhus is approximately 7–10 days [8]. The main clinical features are fever; myalgia; non-pruritic maculopapular or spotted rash on the trunk; and lymphadenopathy. Most patients recover after prompt and proper treatment. However, delayed diagnosis and treatment may lead to bacterium mediated-vascular injury in various vital organs, including the liver, kidneys, meninges and brain [9]; and disseminated intravascular coagulopathy (DIC) with platelet consumption, vascular leakage, pulmonary edema, shock and organ failure, which may eventually result in death [10,11]. 

Scrub typhus is included in a group of acute undifferentiated pyrogenic diseases that encompasses several viral and bacterial diseases, such as leptospirosis, Dengue fever, Hanta virus infection and many others [12,13,14,15]. Along with the patient’s history (e.g., outdoor activity and entering chigger-infested vegetation) and clinical features (the presence of eschar and/or relevant clinical symptoms), confirmation by the standard laboratory methods is required for specific diagnosis of scrub typhus. An indirect immunofluorescence assay (IFA) detects IgM initially, and later IgG antibodies, or a four-fold rise in antibodies against *O. tsutsugamushi* antigen (commonly prepared from a set of Gillian, Karp and Kato strains) in paired acute-convalescent serum samples is the standard diagnostic path [16]. However, the IFA is difficult to perform and requires equipment and expertise. Although an antibody detection assay in the form of rapid tests is available and may be utilized as a point-of-care test, these tests are less sensitive during the early phase of the disease and may not be useful for providing information concerning options for early treatment. Besides, more recently, various versions of PCR with sensitivity ranging from 73 to 100% have been developed for scrub typhus early diagnosis [17,18,19,20]. Nevertheless, PCR is not a point-of-care test, especially for the resource-limited areas where endemic scrub typhus is a local healthcare concern. In this study, we offer an antigen detection test kit (AgTK) in an immunochromatographic (ICT; lateral flow) format for rapid, early and on-site diagnosis of scrub typhus. The assay is easy to perform and has a turn-around time of a few minutes. The highly conserved GroEL chaperonin, a 60 kDa heat shock protein (HSP60), is used as the diagnostic target, as this protein is a predominant antigen that the rickettsial bacteria produce in the infected host [21,22].

## 2. Materials and Methods

### 2.1. Production of Recombinant 60 kDa GroEL of O. tsutsugamushi

The consensus sequence coding for 60 kDa-GRoEL chaperonin (HSP60) of *O. tsutsugamushi* was synthesized commercially (GenScript Biotech, Piscataway, Middlesex County, NJ, USA). For the design of GroEL gene consensus sequence, 32 DNA sequences coding for the GroEL gene of *O. tsutsugamushi* were obtained from the GenBank database and were multiply aligned, and the codons were optimized. The *Eco*RI and *Xho*I restriction sites were added to the 5′ and 3′ ends of the gene sequence, respectively. The DNA was placed in the *T7-tag* and *6*× *His-tag* open reading frame, and synthesized as the pUC57-simple inserted synthetic DNA. The sequences coding for the GroEL were excised from the synthesized plasmids using FastDigest restriction enzymes and ligated to linearized pET23b+ vector backbone using T4 DNA ligase. The recombinant pET23b+ plasmids were introduced into JM109 *E. coli* by using a transformation kit (Thermo Fisher Scientific, Waltham, MA, USA). The inserted DNA was verified by DNA sequencing and the verified recombinant vector was introduced into BL21 (DE3) *E. coli* protein expression host. Appropriate clone of the transformed BL21 (DE3) *E. coli* was grown under 0.4 mM isopropyl-β-D thiogalactopyranoside (IPTG) induction condition and the 6× His tagged-recombinant protein was purified from the bacterial inclusion body (IB) by using a metal affinity resin. In brief, the transformed bacterial cells were lysed by using lysozyme and sonication. The preparation was centrifuged (10,000× *g*, 4 °C, 20 min). Then, the pellet was washed sequentially with Triton X-100 to remove insoluble-trapped proteins; 60% isopropanol to remove endotoxin and detergent; and distilled water (Milli-Q). The purified IB was solubilized with 10% sarkosyl at 25 °C for 24 h and centrifuged as above. The solubilized IB was diluted to 1% sarkosyl and mixed with Triton X-100 and CHAPS before loading to Ni-NTA resin column. The recombinant protein was eluted by using 1% SDS. The excess of SDS in the preparation was removed by cold precipitation. The recombinant protein was subjected to sodium dodecyl sulfate-polyacrylamide gel electrophoresis (SDS-PAGE) and Coomassie Brilliant Blue G-250 (CBB) staining and verified by LC-MS/MS.

### 2.2. Mouse Immunization 

Animal experiments received approval from the Animal Care and Use Committee of The Faculty of Medicine Siriraj Hospital, Mahidol University, Bangkok (SiACUC number 009/2557). Mouse manipulation was performed by a scientist holding a certificate for laboratory animal use certified by the National Research Council of Thailand. Three female BALB/c mice, aged 6 weeks-old, were obtained from The National Laboratory Animal Center, Mahidol University, Salaya campus. They were housed at the animal facility of the Department of Parasitology, the Faculty of Medicine Siriraj Hospital, in a shoe-box type-cage with woodchip bedding placed in air-conditioned room (21–25 °C, 50–70 humidity) under 12-h-light/dark cycle. Feed (mouse pellets) and drinking water were allowed *ad libitum*. Each mouse was immunized intraperitoneally with 10 μg rGroEL in phosphate buffered saline, pH 7.4 (PBS) mixed 3:1 with alum adjuvant (Pierce, Thermo Fisher Scientific) in a total volume of 200 μL. Four booster doses were given to all mice at two weeks-intervals using 20 μg of the same antigen and the same route. Two weeks after the last booster, blood samples were collected from individual mice and the serum antibody titers were determined by using indirect ELISA against the homologous antigen. Western blot patterns of the mouse immune sera against the SDS-PAGE-separated rGroEL were determined.

### 2.3. Production of Mouse Monoclonal Antibodies against the rGroEL 

Conventional hybridoma technique was used to generate the mouse hybridoma secreting monoclonal antibodies for the rGroEL [23]. One of the immunized mice was given an intravenous injection with 20 μg of the rGroEL in 200 μL of PBS. Three days later, the animal was sacrificed humanely; the mouse single spleen cells were prepared and fused with log-phase grown P3x-63-Ag8.653 mouse myeloma cells (10:1) by using 50% PEG-4000 as a fusogen. The fused cells in HAT medium were aliquoted into wells of the 96-wells-tissue culture plates (2 × 10^5^ cells per well). The plates were kept at 37 °C in 5% CO_2_ atmosphere. Spent culture fluids in wells containing growing hybrid cells were replaced appropriately with fresh medium. The culture supernatants of the growing hybrid cells were checked for antibody against the rGroEL by indirect ELISA. The cells in the ELISA-positive wells were cloned by limiting dilution method. Growing hybrids were adapted to serum-free medium and the monoclonal antibodies (mAbs) were typed by using mouse isotyping kit (Thermo Fisher Scientific). Spent medium of the selected hybridoma clone containing mAbs was collected, concentrated and purified by loading onto Protein A column (Pierce™ Protein A, Thermo Fisher Scientific, Rockford, IL, USA) pre-equilibrated with binding buffer (0.02 M sodium phosphate, pH 7.0). The mAb-loaded column was kept on a rotator at 4 °C for 2 h. The resin was washed with 10 column volumes of the buffer; then the column-bound-IgG were eluted out by using 0.1 M glycine-HCl, pH 2.7; and the solution was immediately neutralized with 1 M Tris-HCl, pH 9.0. The mAb preparation was dialyzed against PBS; the protein content was determined and adjusted to 1.5 mg/mL.

### 2.4. Preparation of Rabbit Serum Immune to rGroEL

A New Zealand White rabbit was injected intramuscularly with 2 mL of 125 μg of rGroEL mixed with alum adjuvant. The immunogenic dose was increased to 250 μg in the four booster doses, which were given at 14 day-intervals. Fourteen days after the last booster, the rabbit was bled and the immune serum was collected. Total IgG was purified from the rabbit immune serum by using 50% ammonium sulfate precipitation; then, centrifugation; and finally, the precipitate was dissolved and dialyzed against PBS. The rabbit polyclonal IgG (pAb) was purified as for the mouse mAb described above but using the protein G column (HiTrapTM HP; GE Healthcare Bio-Sciences AB, Uppsala, Sweden). The pAb was dialyzed against PBS; the protein content was determined and adjusted to 1 mg/mL. 

### 2.5. Preparation of Immunochromatographic Antigen Detection Test Kit (ICT AgTK)

The GroEL-specific mAb IgG was used as capture antibody at the ICT test (T) line while the purified rabbit pAb was used in preparing the pAb-colloidal gold conjugate. Full option of the BIODOT XYZ Series machine was used for assembling and preparing the immunochromatographic test strips.

For preparing the pAb-colloidal gold conjugate, the pH of the colloidal gold particles (40 nm in diameter) (Serve Science, Bangkok, Thailand) was adjusted to 8.2. The pAb (100 μg in 100 μL PBS) was added to 20 mL of the gold suspension and kept at room temperature (25 °C) on a slow rocker for 5 min. The unoccupied sites on the surface of the gold particles were blocked by mixing with 1 mL of 10% bovine serum albumin (BSA) at room temperature on the rocker for 1 h. The preparation was centrifuged at 9200× *g*, 25 °C for 30 min. The pellet was re-suspended in 1 mL of the gold storage buffer (1% BSA in 2 mM borate buffer, pH 8.2). OD 540 nm of the pAb-colloidal gold conjugate was adjusted to 10 before impregnating into the conjugate pad of the ICT.

The GroEL-specific mAb (1.5 mg/mL) and 0.5 mg/mL anti-rabbit GAR antibody (Lampire, Pipersville, PA, USA) were sprayed on a nitrocellulose membrane (FF60/100; Whatman, Wiltshire, Marlborough, MA, USA) at 5 mm-interval between the two lines, designated Test line (T) and Control line (C), respectively. The sample pad, the conjugate pad, the nitrocellulose membrane with the T and C lines and the reagent absorbent pad were assembled appropriately on a laminate backing card. The assembled sheet was cut into strips of 4 mm-width. They were then placed individually into plastic cassettes, or used without cassettes as lateral flow strip tests.

### 2.6. Clinical Samples

Three sets of clinical samples were used in this study. The first sample set were 40 deep-frozen serum samples of patients with febrile illnesses from the collection of the Department of Medicine, Faculty of Medicine Siriraj Hospital, Mahidol University, Bangkok. The second set of samples were fresh plasma and the respective buffy coat samples from 15 patients with fever of Nan Provincial Hospital, located at about 670 km north of Bangkok. The third sample set were 20 serum samples of patients with fever who seek treatment at Prasart Hospital, Surin Province, located about 435 km northeast of Bangkok. The use of human samples was approved by the Institutional Review Board (IRB) of the Faculty of Medicine Siriraj Hospital, Mahidol University (number 009/2557).

### 2.7. Sample Testing by ICT AgTK

For testing the samples by using the ICT AgTK developed in this study, 10 μL of each sample (serum or plasma or buffy coat) were mixed with 100 μL of running buffer (10 mM phosphate buffer, 100 mM NaCl, 0.05% Triton X-100, pH 7.4) for 1 min before applying to the sample pad of the ICT AgTK. The result was read 15 min after the sample application. Two red lines at the C and T indicated positive reaction (the presence of GroEL in the sample), while only one red line at the C indicated negative reaction. 

The medical diagnoses associated with each sample were initially masked from our laboratory. Analysis of sample sets one and two were performed in our laboratory. The results were then sent back to the sample sources where collaborators could unmask and reveal the original medical diagnoses associated with each sample. Original diagnoses were based on the patients’ history; the patients’ clinical features; and laboratory tests, including *Leptospira* IFA for leptospirosis and *O.* *tsutsugamushi* IFA for scrub typhus using test kits from the Department of Medical Sciences, Ministry of Public Health, Thailand, and Dengue NS1 antigen and Dengue IgG/IgM ELISA (SD Biosensor, Suwon-si, Gyeonggi-do, Korea) for Dengue fever.

For independent laboratory validation, the ICT AgTK was sent to the Clinical Pathology Laboratory of Prasat Hospital. The test kit validation was utilized by a professional medical technologist in comparison with the clinical features and the test kits (SD Biosensor, Korea) used routinely at the hospital.

### 2.8. Real-Time Polymerase Chain Reaction

SYBR 202 real-time PCR assays to detect scrub typhus were also used for comparison with the ICT AgTK. DNA was extracted from individual plasma samples of Nan Provincial hospital using Bacteria Genomic DNA Kit (Presto™ Mini gDNA Bacteria Kit, No. GBB100, Geneaid Biotech, Shilr District, New Taipei City, Taiwan) following the manufacturer’s instructions. Real-time PCR could not be used for analyses of the first or third sample sets. SYBR real-time PCR assays were used for the specific detection of the gene coding for the GroEL of *O. tsutsugamushi.* The primer pair were: F: TAA TTG CTA GTG CAA TGT CTG CGT T and R: CCA AAG TCA CGA TCA GCT ATA CT. The PCR reaction mixture contained 200 nM each primer, 1 µL of DNA template, 10 µL of master mix (KAPA SYBR FAST qPCR Master Mix) containing SYBR green, *Taq* polymerase, 4 mM MgCl_2_, dNTPs and distilled water to a final volume of 20 µL. The PCR reactions were carried out and analyzed using real-time thermocycler (CFX96 Touch Real-Time PCR Detection System, BioRad, Hercules, CA, USA) with an initial temperature of 95 °C for 3 min, followed by 40 cycles at 95 °C for 3 s, 55 °C for 30 s and 72 °C for 20 s, with fluorescence monitoring at the 55 °C annealing step on a predetermined SYBR channel. Melting curve analysis was performed with increment of 1 °C/step (72–95 °C) to determine the change in peak fluorescence over time (dF/dT). 

### 2.9. Statistical Analysis

Diagnostic sensitivity, specificity, accuracy and positive and negative predictive values of the ICT AgTK in comparison to the original diagnoses were determined [24]. 

## 3. Results

### 3.1. Recombinant GroEL of O. tsutsugamushi 

**Figure 1:**
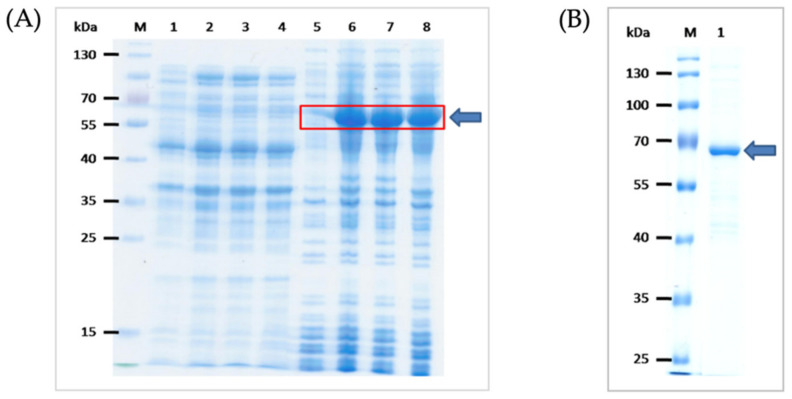
Recombinant GroEL preparation. (**A**) SDS-PAGE-separated proteins in the soluble and insoluble fractions of transformed BL21 (DE) *E. coli* carrying pET23b+ with inserted DNA coding for rGroEL of *O. tsutsugamushi*. Lanes 1 and 5, proteins in soluble and insoluble fractions of original BL21 (DE3) *E. coli*, respectively; lanes 2–4, proteins in the soluble fractions of transformed BL21 (DE3) *E. coli*, clones 1–3, respectively; lanes 6–8, proteins in the insoluble fractions of transformed BL21 (DE3) *E. coli*, clones 1–3, respectively. The recombinant protein was predominant in the *E. coli* insoluble fraction (red box and arrow). (**B**) Purified recombinant protein (arrow) from the transformed *E. coli* insoluble fraction solubilized in 1% SDS. Lanes M of (**A**,**B**) are protein molecular weight marker. Numbers on the left of both Figures are protein masses in kDa.

Multiple alignments of the 60 kDa GroEL chaperonin sequences from different *O. tsutsugamushi* strains and the consensus amino acid sequence are shown in Figure S1. The SDS-PAGE-separated proteins in the soluble and insoluble fractions of transformed BL21 (DE3) *E. coli* carrying pET23b+ with inserted DNA coding for rGroEL of *O. tsutsugamushi* and the purified recombinant protein are shown in Figure 1. The recombinant protein was verified as *O. tsutsugamushi* GroEL chaperonin by LC-MS/MS (data not shown).

### 3.2. Rabbit and Mouse Sera Immune to rGroEL 

**Figure 2:**
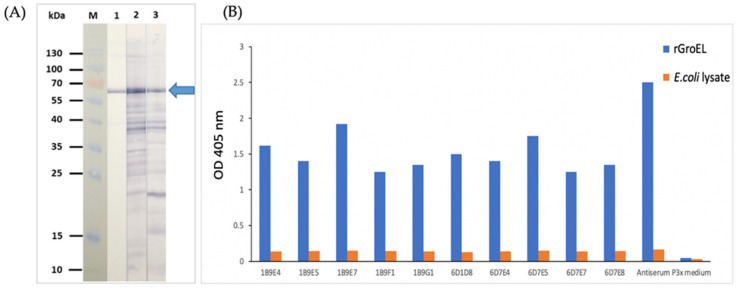
Mouse and rabbit sera immune to rGroEL and mouse hybridomas secreting monoclonal antibodies against rGroEL. (**A**) Western blot patterns of the mouse and rabbit polyclonal antibodies that bound to the SDS-PAGE-separated rGroEL of *O. tsutsugamushi.* Lane M, protein molecular mass marker; lane 1, positive control, i.e., the SDS-PAGE-separated rGroEL probed with anti-His monoclonal antibody; lane 2, SDS-PAGE-separated rGroEL probed with serum of immunized mouse number 1; lane 3, SDS-PAGE-separated rGroEL probed with rabbit immune serum. Numbers on the left are protein masses in kDa. The rGroEL-antibody reactive bands are indicated by arrow. (**B**) OD 405 nm of indirect ELISA for testing binding of monoclonal antibodies in culture supernatants of 10 hybridomas to purified rGroEL in comparison with the control antigen (lysate of original BL21 (DE3) *E. coli* host). Mouse serum immune to rGroEL (Antiserum) and culture supernatant of the P3x-63-Ag8.653 mouse myeloma cells P3x medium) were included as positive and negative binding controls.

All BALB/c mice immunized intraperitoneally with the rGroEL mixed with alum had the serum titer at 1:512,000. Likewise, the rabbit immunized intramuscularly with rGroEL mixed with alum adjuvant had serum ELISA titer against the homologous antigen at 1: 512,000. Western blot analysis revealed that the mouse and rabbit immune sera contained antibodies for the rGroEL (Figure 2A).

### 3.3. Mouse Monoclonal Antibodies against rGroEL

From the fusion of 1.62 × 10^8^ immune splenocytes of the rGroEL-immunized mouse and the P3x-63-Ag8.653 mouse myeloma cells (10:1), a total of 10 growing hybrid cells (hybrodomas) secreted antibodies that bound to rGroEL by indirect ELISA were obtained (Figure 2B).

### 3.4. Detection Limit of the ICT AgTK 

**Figure 3:**
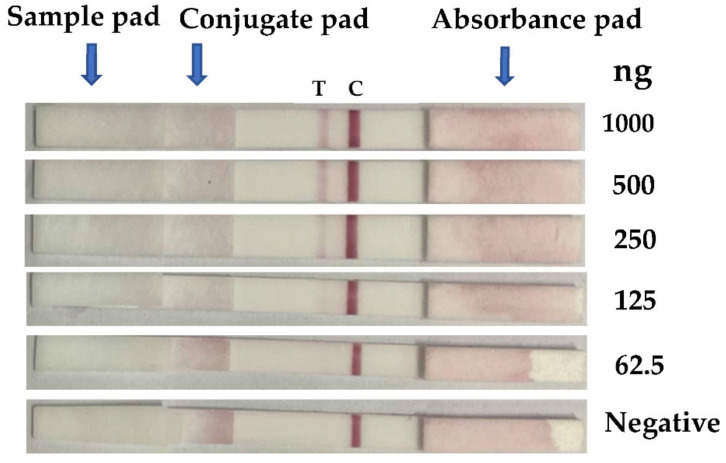
The rGroEL detection limit of the ICT AgTK. Different amounts of rGroEL (62.5–1000 ng) in 10 µL PBS were applied to the ICT AgTK. Negative was PBS alone. The lowest amount of the rGroEL that could be detected by the test kit was 125 ng. The results of the ICT AgTK were read 5 min after applying the samples. A positive result is seen as two red lines (the left line is the test (T) line, and the right line is the control (C) line); only C shows a negative result.

The ICT AgTK prepared as described in Section 2.5 were tested for the limit of rGroEL detection. Ten microliter aliquots of PBS containing different amounts of rGroEL (62.5–1000 ng) were applied to the ICT AgTK. The smallest amount of the rGroEL that could be detected was 125 ng (Figure 3).

### 3.5. Performance of the ICT AgTK on Clinical Samples in Comparison to the Combined Clinical and Standard IFA Diagnoses

In-house validation of the ICT AgTK revealed that among the 40 frozen sera of the first set samples (Table S1) that were tested blindly, 17 samples (numbers 2–10, 11–17, 19 and 20) were positive for scrub typhus; the remaining 23 samples were negative according to the ICT AgTK (Table S1). After completion of the test, the ICT AgTK test results were sent for unmasking of the original diagnoses for the original serum sources. It was found that the 17 samples that were tested positive by the ICT AgTK were also positive for antibodies against *O. tsutsugamushi* according to the standard IFA (Table S1). Among the 23 samples tested negative by the ICT AgTK, 3 samples (numbers 1, 11 and 18) were positive for scrub typhus antibody via the IFA (Table S1). Of the other 20 samples that were found negative by the ICT AgTK, 10 samples (numbers 21–30) were found positive for antibodies against Dengue virus NS1 protein using the SD Bioline antigen detection test kit, but negative for IgM and IgG for Dengue fever, leptospirosis and scrub typhus by the respective IFA assays (Table S1). Serum samples 31–40 were positive for either IgM or IgG for leptospirosis but negative for anti-Dengue NS1 and Dengue antibodies and scrub typhus antibodies by IFA (Table S1). The ICT AgTK for detecting the GroEL of *O. tsutsugamushi* in the frozen serum samples showed 85% diagnostic sensitivity, 100% diagnostic specificity and 92.5% accuracy, when compared with the clinical diagnosis and standard IFA. The positive and negative predictive values of ICT AgTK were 100 and 86.95%, respectively.

The fresh plasma and buffy coat samples of patients from Nan Hospital (Table S2) were evaluated blindly by using ICT AgTK and real-time PCR (performed by different scientists). The melting curve of the real-time PCR for detecting the gene coding for *O. tsutsugamushi* GroEL is shown in Figure S2. The results were then compared to the clinical and IFA results performed at Nan Hospital. Ten of the fresh 15 plasma and buffy coat samples from Nan Province were positive according to the ICT AgTK, and they were also positive by the real-time PCR (Table S2). These positive samples were originally from patients diagnosed with scrub typhus based on the clinical features and IFA tests performed at Nan Hospital’s laboratory. The five plasma samples that were negative by the ICT AgTK and the real-time PCR were originally from Dengue fever-diagnosed patients (three plasma samples) and leptospirosis-diagnosed patients (two plasma samples) (Table S2). Thus, the results of the ICT AgTK were consistent with those obtained by real-time PCR and results diagnosed originally found by IFA at Nan Hospital.

The ICT AgTK was evaluated independently at Prasat Hospital’s laboratory, Surin Province by the hospital’s medical technologist, in comparison with the results of commercialized test kit for detection of antibody (IgM/IgG) against *O. tsutsugamushi*. Among 20 serum samples that were tested, six scrub typhus samples out of 20 serum samples were positive using the ICT AgTK kits (five strongly-positive; one gave a faint band at the test line); the remaining 14 samples were negative (Table S3). The ICT AgTK results were completely consistent with the results of IgM/IgG antibody detection by the commercial IFA test.

## 4. Discussion

No effective vaccine against scrub typhus is available, although developing one is an imperative need, as the endemic area of scrub typhus is expanding and the global healthcare burden caused by the disease is increasing [25]. Scrub typhus may result in a high mortality rate if left untreated [3]. Therefore, early and proper diagnosis together with prompt treatment are absolute necessities for preventing the fatal complications associated with the disease [26,27]. In the absence of the pathognomonic eschar, scrub typhus is mainly diagnosed based on the patient’s history, clinical features and laboratory testing, which involves serological assays (antibody detection) such as indirect immunofluorescence assay (IFA) [28]. At present, no point-of-care diagnostics for scrub typhus are available. In this study, an antigen detection test kit (AgTK) based on the lateral flow technology (immunochromatography, ICT) was developed for rapid, early, simple and on-site scrub typhus diagnosis. To our knowledge, this is the first scrub typhus diagnostic in the form of an antigen detection test kit.

Most serological assays for detecting antibodies against *O. tsutsugamushi* in the clinical samples of patients suspected of scrub typhus use a set of standard antigens prepared from Karp, Kato and Gilliam prototypic strains. However, for many isolated *Orientia tsutsugamushi* strains, their antigenicity and virulence are different from the prototypic strains. Many such strains have been reported in various parts of Asia, especially Southeast Asia, i.e., strains TA 678, TA686, TA716, TA763 and TH1817 [29,30,31,32]. The *O. tsutsugamushi* Southeast Asian isolates could be classified serologically into at least 29 antigenic types [33], reflecting the high antigenic diversity of *O. tsutsugamushi*. The diversity is contributed mainly by the O antigens, e.g., 56 and 70 kDa surface-exposed proteins [21]. The strain diversity may render false negative results for the antibody detection assay. Besides, the antibody detection is known to have low sensitivity in the early phase of the infection. 

The 60 kDa GroEL or heat shock protein 60 (HSP60) is one of molecular chaperones of *O. tsutsugamushi*. Like other chaperone proteins, the GroEL is pivotal not only for facilitating the normal folding and assembly of many proteins and catalyzing the proteolytic degradation of abnormal proteins, but also for the bacterial growth and survival, particularly in hostile environments such as the intracellular milieu [21,34]. *O. tsutsugamushi* GroEL is a highly immunogenic protein actively produced by the rickettsial bacteria in the infecting host [21]. The protein is highly conserved among members of the genus *Orientia* [21]. The overall nucleotide identity between all available *O. tsutsugamushi* isolates is 98.8% (range: 95.0–100), whereas the nucleotide identities between *O. tsutsugamushi* and typhus and spotted fever rickettsiae are only 67.5 and 65.6%, respectively [35]. Therefore, we exploit the highly conserved *O. tsutsugamushi* GroEL-chaperonin contained in blood-derived samples of the patients, either serum, plasma or buffy coat samples as the diagnostic target of the AgTK for scrub typhus early diagnosis.

Even though the *O. tsutsugamushi* GRoEL is highly conserved, in this study we produced the *E. coli* derived-recombinant GroEL expressed from the consensus gene sequence obtained from a multiple alignment of 32 *O. tsutsugamushi* GroEL sequences extracted from the GenBank database. The rGroEL was used as the immunogen in the mouse immunization for the production of mouse monoclonal antibodies that were used for preparing the antigen detection reagent in the ICT test line. This is to ensure that the antibodies, especially the monoclonal antibodies that were used as the GroEL detection reagent at the ICT test line will bind to the GroEL epitope contained in all prevailing strains and variants of *O. tsutsugamushi*.

When the ICT AgTK was used to examine frozen serum samples that had been kept for many months/years, a decrease in diagnostic efficacy was observed compared to use of the test kits on fresh plasma and buffy coat samples (from Nan Province), or fresh serum samples (Prasat Hospital). This should be due to the decay of the target antigenic protein. In common clinical practice, however, fresh samples, either serum, plasma or buffy coat can be obtained from the patients on the day that they seek treatment. Such samples could be applied directly to the test kit, negating any affects on test kit performance. Unfortunately, performance of the test kits using fresh whole blood samples was not tested. Another limitation of this study is the numbers of tested fresh samples were not many. This was because the cases of scrub typhus in Thailand are sporadic. The best way to test the AgTK efficacy on high case numbers is to send the test kit to the clinical pathology laboratories of many provincial hospitals for validation on fresh samples which will take long and unpredictable time. For the time being, the overall results in this study indicate that the so-developed ICT AgTK can be used for point-of-care scrub typhus diagnosis particularly in the resource-limited areas where there are high incidences of undifferentiated febrile illnesses that require specific and differential diagnoses.

## 5. Conclusions

Delayed diagnosis and treatment of scrub typhus may lead to death due to severe complications. In this study, an antigen detection test kit (AgTK) in the format of immunochromatography (ICT) was developed for rapid and early diagnosis of scrub typhus using the highly conserved 60 kDa GroEL chaperonin of *O. tsutsugamushi* as the diagnostic target. In-house validation by using 40 frozen serum samples of patients with pyrogenic infections revealed that the ICT AgTK has 85, 100 and 95% diagnostic sensitivity, specificity and accuracy, respectively, when evaluated using the combined clinical features of patients and standard IFA. When tested on fresh samples, however, the test kits correctly identified 10 scrub typhus out of 15 fresh plasma/buffy coat samples of patients with febrile illnesses. Independent laboratory validation showed that the test kit correctly identified six scrub typhus out of 20 serum samples of patients with fever, consistent with the specific IgM/IgG detection by standard IFA. The ICT AgTK is easy to perform and has a rapid turn-around time. It should be used for on-site and early scrub typhus diagnosis, especially in resource-limited healthcare settings, by using the freshly collected samples, either serum, or plasma, or buffy coat.

## Data Availability

The data sets used during current study are available from the corresponding author on reasonable request.

## Supplementary Materials

The following are available online at https://www.mdpi.com/article/10.3390/diagnostics12010136/s1. Figure S1: CLUSTAL Omega (1.2.0) multiple sequence alignment of GroEL chaperonin of O. tsutsugamushi and the consensus sequence. Figure S2: Melting curve of real-time PCR for detecting gene coding for O. tsutsugamushi GroEL. Table S1: Performance of the ICT AgTK on the 40 frozen serum samples compared with the original diagnoses based on clinical features and standard IFA and/or detection of anti-Dengue virus NS1 by using SD Bioline antigen detection kit. Table S2: Results of the GRoEL detection by using the ICT AgTK and real-time PCR performed blindly in our laboratory in comparison to the O. tsutsugamushi IgM/IgG IFA performed at the laboratory of Nan Hospital. Table S3: Performance of the ICT AgTK evaluated independently at Prasat Hospital, Surin Province, by the hospital’s medical technologist in comparison with the results of commercialized test kit for detection of antibody (IgM/IgG) against O. tsutsugamushi.
