# Supplementary material for: GroEL Chaperonin-Based Assay for Early Diagnosis of Scrub Typhus
Source: Diagnostics (Basel). 2022 Jan 6;12(1):136. doi: 10.3390/diagnostics12010136 (PMC8775097; doi:10.3390/diagnostics12010136)
Supplement: Supplementary file 1 [file diagnostics-12-00136-s001.zip › diagnostics-1436134-SI.pdf]

Supplementary data

GroEL Chaperonin-Based Assay for Early Diagnosis of Scrub Typhus

Nitaya Indrawattana [1], Pisinee Aiumurai [2], Nawannaporn Sae-lim [2], Watee Seesuay [2], Onrapak Reamtong [3], Manas Chongsa-nguan [1,4], Wanpen Chaicumpa [2] and Nitat Sookrung [2,5,*]

[1] Department of Microbiology and Immunology, Faculty of Tropical Medicine, Mahidol University,
Bangkok 10400, Thailand; nitaya.ind@mahidol.ac.th (N.I.)

[2] Center of Research Excellence on Therapeutic Proteins and Antibody Engineering, Department of Parasitolgy, Faculty of Medicine Siriraj Hospital, Mahidol University, Bangkok 10700, Thailand; pisinee.aiu@mahidol.ac.th (P.A.-u); nawannaporn.sae@mahidol.edu (N.S.); watee.see@gmail.com (W.S.); wanpen.cha@mahidol.ac.th (W.C.)

[3] Department of Molecular Tropical Medicine and Genetics, Faculty of Tropical Medicine, Mahidol University, Bangkok 10400, Thailand; onrapak.rea@mahidol.ac.th

[4] Faculty of Public Health and Environment, Pathumthani University, Pathum Thani 12000; Thailand, mchongsanguan@yahoo.com (M.C.-n)

[5] Biomedical Research Incubator Unit, Department of Research, Faculty of Medicine Siriraj Hospital,
Mahidol University, Bangkok 10700, Thailand

* Correspondence: nitat.soo@mahidol.ac.th; Tel.: +66-2-4196491

```
AY191588.1      ------------------------------------------------------------
AY191589.1      ------------------------------------------------------------
EF551310.1      ------------------------------------------------------------
EF551303.1      ------------------------------------------------------------
AY191586.1      ------------------------------------------------------------
CAM79983.1      MSKQIVHSDQCRKKIIEGINVVANAVGITLGPKGRCVAIEQSYGPPKITKDGVSVAKAIQ
BAG39717.1      MSKQIVHGDQCRKKIIEGINVVANAVGITLGPKGRCVAIEQSYGPPKITKDGVSVAKAIQ
M31887.1        MSKQIVHGDQCRKKIIEGINVVANAVGITLGPKGRCVAIEQSYGPPKITKDGVSVAKAIQ
EF551292.1      ------------------------------------------------------------
EF551295.1      ------------------------------------------------------------
EF551296.1      ------------------------------------------------------------
EF551297.1      ------------------------------------------------------------
EF551298.1      ------------------------------------------------------------
EF551299.1      ------------------------------------------------------------
EF551301.1      ------------------------------------------------------------
EF551302.1      ------------------------------------------------------------
EF551304.1      ------------------------------------------------------------
EF551306.1      ------------------------------------------------------------
EF551307.1      ------------------------------------------------------------
EF551309.1      ------------------------------------------------------------
EF551290.1      ------------------------------------------------------------
EF551293.1      ------------------------------------------------------------
EF551294.1      ------------------------------------------------------------
EF551300.1      ------------------------------------------------------------
EF551305.1      ------------------------------------------------------------
EF551308.1      ------------------------------------------------------------
EF551289.1      ------------------------------------------------------------
EF551291.1      ------------------------------------------------------------
AY191585.1      ------------------------------------------------------------
EF551288.1      ------------------------------------------------------------
AY059015.1      ------------------------------------------------------------
AY191587.1      ------------------------------------------------------------

                MSKQIVHSDQCRKKIIEGINVVANAVGITLGPKGRCVAIEQSYGPPKITKDGVSVAKAIQ

AY191588.1      ------------------------------------------------------------
AY191589.1      ------------------------------------------------------------
EF551310.1      ------------------------------------------------------------
EF551303.1      ------------------------------------------------------------
AY191586.1      ------------------------------------------------------------
CAM79983.1      LKDKSLNVGAQFVISVASKTADVAGDGTTTATVIADAAVRELNKAEVAGIDIQEVRKGAE
BAG39717.1      LKDKSLNVGAQFVISVASKTADVAGDGTTTATVIADAAVRELNKAEVAGIDIQEVRKGAE
M31887.1        LKDKSLNVGAQFVISVASKTADVAGDGTTTATVIADAAVRELNKAEVAGIDIQEVRKGAE
EF551292.1      ------------------------------------------------------------
EF551295.1      ------------------------------------------------------------
EF551296.1      ------------------------------------------------------------
EF551297.1      ------------------------------------------------------------
EF551298.1      ------------------------------------------------------------
EF551299.1      ------------------------------------------------------------
EF551301.1      ------------------------------------------------------------
EF551302.1      ------------------------------------------------------------
EF551304.1      ------------------------------------------------------------
EF551306.1      ------------------------------------------------------------
EF551307.1      ------------------------------------------------------------
EF551309.1      ------------------------------------------------------------
EF551290.1      ------------------------------------------------------------
EF551293.1      ------------------------------------------------------------
EF551294.1      ------------------------------------------------------------
EF551300.1      ------------------------------------------------------------
EF551305.1      ------------------------------------------------------------
EF551308.1      ------------------------------------------------------------
EF551289.1      ------------------------------------------------------------
EF551291.1      ------------------------------------------------------------
AY191585.1      ------------------------------------------------------------
EF551288.1      ------------------------------------------------------------
AY059015.1      ------------------------------------------------------------
AY191587.1      ------------------------------------------------------------
```

```
                 LKDKSLNVGAQFVISVASKTADVAGDGTTTATVIADAAVRELNKAEVAGIDIQEVRKGAE

AY191588.1       ------------------------------------------------------------
AY191589.1       ------------------------------------------------------------
EF551310.1       ------------------------------------------------------------
EF551303.1       ------------------------------------------------------------
AY191586.1       ------------------------------------------------------------
CAM79983.1       KAVEAVIADVRKNSSPVKNEEEIAQVATVSSNGDREIGEKIANAMKQVGQEGVITVEDSK
BAG39717.1       KAVEAVIADVRKNSSPVKNEEEIAQVATVSSNGDREIGEKIANAMKQVGQEGVITVEDSK
M31887.1         KAVEAVIADVRKNSSPVKNEEEIAQVATVSSNGDREIGEKIANAMKQVGQEGVITVEDSK
EF551292.1       ------------------------------------------------------------
EF551295.1       ------------------------------------------------------------
EF551296.1       ------------------------------------------------------------
EF551297.1       ------------------------------------------------------------
EF551298.1       ------------------------------------------------------------
EF551299.1       ------------------------------------------------------------
EF551301.1       ------------------------------------------------------------
EF551302.1       ------------------------------------------------------------
EF551304.1       ------------------------------------------------------------
EF551306.1       ------------------------------------------------------------
EF551307.1       ------------------------------------------------------------
EF551309.1       ------------------------------------------------------------
EF551290.1       ------------------------------------------------------------
EF551293.1       ------------------------------------------------------------
EF551294.1       ------------------------------------------------------------
EF551300.1       ------------------------------------------------------------
EF551305.1       ------------------------------------------------------------
EF551308.1       ------------------------------------------------------------
EF551289.1       ------------------------------------------------------------
EF551291.1       ------------------------------------------------------------
AY191585.1       ------------------------------------------------------------
EF551288.1       ------------------------------------------------------------
AY059015.1       ------------------------------------------------------------
AY191587.1       ------------------------------------------------------------

                 KAVEAVIADVRKNSSPVKNEEEIAQVATVSSNGDREIGEKIANAMKQVGQEGVITVEDSK

AY191588.1       -----VEVVKGMRFDRGYISQYFATNREKMITEFENPYILLLDQKVSAVQPLVPILQAVS
AY191589.1       -----VEVVKGMRFDRGYISQYFATNREKMITEFENPYILLLDQKVSTVQPLVPVLEAVA
EF551310.1       -------------------SQYFATNREKMITEFENPYILLLDQKISTVQPLVPVLEAVA
EF551303.1       -------------------SQYFATNREKMITEFENPYILLLDQKVSTXQPLVPVLEAVA
AY191586.1       -----VEVVKGMRFDRGYISQYFATNREKMITEFENPYILLLDQKVSTVQPLVPVLEAVA
CAM79983.1       NFNFEVEVVKGMRFDRGYISQYFATNREKMITEFENPYILLLDQKVSTVQPLVPVLEAVA
BAG39717.1       NFNFEVEVVKGMRFDRGYISQYFATNREKMITEFENPYILLLDQKVSTVQPLVPVLEAVA
M31887.1         NFNFEVEVVKGMRFDRGYISQYFATNREKMITEFENPYILLLDQKVSTVQPLVPVLEAVA
EF551292.1       -------------------SQYFATNREKMITEFENPYILLLDQKVSTVQPLVPVLEAVA
EF551295.1       -------------------SQYFATNREKMITEFENPYILLLDQKVSTVQPLVPVLEAVA
EF551296.1       -------------------SQYFATNREKMITEFENPYILLLDQKVSTVQPLVPVLEAVA
EF551297.1       -------------------SQYFATNREKMITEFENPYILLLDQKVSTVQPLVPVLEAVA
EF551298.1       -------------------SQYFATNREKMITEFENPYILLLDQKVSTVQPLVPVLEAVA
EF551299.1       -------------------SQYFATNREKMITEFENPYILLLDQKVSTVQPLVPVLEAVA
EF551301.1       -------------------SQYFATNREKMITEFENPYILLLDQKVSTVQPLVPVLEAVA
EF551302.1       -------------------SQYFATNREKMITEFENPYILLLDQKVSTVQPLVPVLEAVA
EF551304.1       -------------------SQYFATNREKMITEFENPYILLLDQKVSTVQPLVPVLEAVA
EF551306.1       -------------------SQYFATNREKMITEFENPYILLLDQKVSTVQPLVPVLEAVA
EF551307.1       -------------------SQYFATNREKMITEFENPYILLLDQKVSTVQPLVPVLEAVA
EF551309.1       -------------------SQYFATNREKMITEFENPYILLLDQKVSTVQPLVPVLEAVA
EF551290.1       -------------------SQYFATNREKMITEFENPYILLLDQKVSTVQPLVPVLEAVA
EF551293.1       -------------------SQYFATNREKMITEFENPYILLLDQKVSTVQPLVPVLEAVA
EF551294.1       -------------------SQYFATNREKMITEFENPYILLLDQKVSTVQPLVPVLEAVA
EF551300.1       -------------------SQYFATNREKMITEFENPYILLLDQKVSTVQPLVPVLEAVA
EF551305.1       -------------------SQYFATNREKMITEFENPYILLLDQKVSTVQPLVPVLEAVA
EF551308.1       -------------------SQYFATNREKMITEFENPYILLLDQKVSTVQPLVPVLEAVA
EF551289.1       -------------------SQYFATNREKMITEFENPYILLLDQKVSTVQPLVPVLEAVA
EF551291.1       -------------------SQYFATNREKMITEFENPYILLLDQKVSTVQPLVPVLEAVA
AY191585.1       -----VEVVKGMRFDRGYISQYFATNREKMITEFENPYILLLDQKVSTVQPLVPVLEAVA
EF551288.1       -------------------SQYFATNREKMITEFENPYILLLDQKVSTVQPLVPVLEAVA
```

```
AY059015.1        -----VEVVKGMRFDRGYISQYFATNREKMITEFENPYILLLDQKVSTVQPLVPVLEAVA
AY191587.1        -----VEVVKGMRFDRGYISQYFATNREKMITEFENPYILLLDQKVSTVQPLVPVLEAVA
                                     **************************:*: *****:*:**:
                  NFNFEVEVVKGMRFDRGYISQYFATNREKMITEFENPYILLLDQKVSTVQPLVPVLEAVA

AY191588.1        DTGKPLVLIADDVDGEALTALILNNLKGSIKVVAVKAPGFGDRKKEMLEDIAILTNGEVI
AY191589.1        HTGKPLELIADDVDGEALTALILNNLKGSIKVVAVKAPGFGDRKKEMLEDIAILTNGEVI
EF551310.1        HTGKPLVLIADDVDGEALTALILNNLKGSIKVVAVKAPGFGDRKKEMLEDIAILTNGEVI
EF551303.1        HTGKPLVLIADDVDGEALTALILNNLKGSIKVVAVKAPGFGDRKKEMLEDIAILTNGEVI
AY191586.1        HTGKPLVLIADDVDGEALTALILNNLKRSIKVVAVKAPGFGDRKKEMLEDIAILTNGEVI
CAM79983.1        HTGKPLVLIADDVDGEALTALILNNLKGSIKVVAVKAPGFGDRKKEMLEDIAILTNGEVI
BAG39717.1        HTGKPLVLIADDVDGEALTALILNNLKGSIKVVAVKAPGFGDRKKEMLEDIAILTNGEVI
M31887.1          HTGKPLVLIADDVDGEALTALILNNLKGSIKVVAVKAPGFGDRKKEMLEDIAILTNGEVI
EF551292.1        HTGKPLVLIADDVDGEALTALILNNLKGSIKVVAVKAPGFGDRKKEMLEDIAILTNGEVI
EF551295.1        HTGKPLVLIADDVDGEALTALILNNLKGSIKVVAVKAPGFGDRKKEMLEDIAILTNGEVI
EF551296.1        HTGKPLVLIADDVDGEALTALILNNLKGSIKVVAVKAPGFGDRKKEMLEDIAILTNGEVI
EF551297.1        HTGKPLVLIADDVDGEALTALILNNLKGSIKVVAVKAPGFGDRKKEMLEDIAILTNGEVI
EF551298.1        HTGKPLVLIADDVDGEALTALILNNLKGSIKVVAVKAPGFGDRKKEMLEDIAILTNGEVI
EF551299.1        HTGKPLVLIADDVDGEALTALILNNLKGSIKVVAVKAPGFGDRKKEMLEDIAILTNGEVI
EF551301.1        HTGKPLVLIADDVDGEALTALILNNLKGSIKVVAVKAPGFGDRKKEMLEDIAILTNGEVI
EF551302.1        HTGKPLVLIADDVDGEALTALILNNLKGSIKVVAVKAPGFGDRKKEMLEDIAILTNGEVI
EF551304.1        HTGKPLVLIADDVDGEALTALILNNLKGSIKVVAVKAPGFGDRKKEMLEDIAILTNGEVI
EF551306.1        HTGKPLVLIADDVDGEALTALILNNLKGSIKVVAVKAPGFGDRKKEMLEDIAILTNGEVI
EF551307.1        HTGKPLVLIADDVDGEALTALILNNLKGSIKVVAVKAPGFGDRKKEMLEDIAILTNGEVI
EF551309.1        HTGKPLVLIADDVDGEALTALILNNLKGSIKVVAVKAPGFGDRKKEMLEDIAILTNGEVI
EF551290.1        HTGKPLVLIADDVDGEALTALILNNLKGSIKVVAVKAPGFGDRKKEMLEDIAILTNGEVI
EF551293.1        HTGKPLVLIADDVDGEALTALILNNLKGSIKVVAVKAPGFGDRKKEMLEDIAILTNGEVI
EF551294.1        HTGKPLVLIADDVDGEALTALILNNLKGSIKVVAVKAPGFGDRKKEMLEDIAILTNGEVI
EF551300.1        HTGKPLVLIADDVDGEALTALILNNLKGSIKVVAVKAPGFGDRKKEMLEDIAILTNGEVI
EF551305.1        HTGKPLVLIADDVDGEALTALILNNLKGSIKVVAVKAPGFGDRKKEMLEDIAILTNGEVI
EF551308.1        HTGKPLVLIADDVDGEALTALILNNLKGSIKVVAVKAPGFGDRKKEMLEDIAILTNGEVI
EF551289.1        HTGKPLVLIADDVDGEALTALILNNLKGSIKVVAVKAPGFGDRKKEMLEDIAILTNGEVI
EF551291.1        HTGKPLVLIADDVDGEALTALILNNLKGSIKVVAVKAPGFGDRKKEMLEDIAILTNGEVI
AY191585.1        HTGKPLVLIADDVDGEALTALILNNLKGSIKVVAVKAPGFGDRKKEMLEDIAILTNGEVI
EF551288.1        HTGKPLVLIADDVDGEALTALILNNLKGSIKVVAVKAPGFGDRKKEMLEDIAILTNGEVI
AY059015.1        HTGKPLVLIADDVDGEALTALILNNLKGSIKVVAVKAPGFGDRKKEMLEDIAILTNGEVI
AY191587.1        HTGKPLVLIADDVDGEALTALILNNLKGSIKVVAVKAPGFGDRKKEMLEDIAILTNGEVI
                  .***** ******************** ********************************
                  HTGKPLVLIADDVDGEALTALILNNLKGSIKVVAVKAPGFGDRKKEMLEDIAILTNGEVI

AY191588.1        TEQLGIKLEKVNDTSKLGTANRVIVTKDHTTIVHDKNNSDIEKKVNSRCEQIREAIKDTT
AY191589.1        TEQLGIKLEKVNDTSKLGTANRVIVTKDHTTIVHDKNNSDIEKKVNSRCEQIREAIKDTT
EF551310.1        TEQLGIKLEKVNDTSKLGTANRVIVTKDHTTIVHDKNNSDIEKKVNSRCEQ---------
EF551303.1        TEQLGIKLEKVNDTSKLGTANRVIVTKDHTTIVHDKNNSDIEKKVNSRCEQ---------
AY191586.1        TEQLGIKLEKVNDTSKLGTANRVIVTKDHTTIVHDKNNSDIEKKVNSRCEQIREAIKDTT
CAM79983.1        TEQLGIKLEKVNDTSKLGTANRVIVTKDHTTIVHDKNNSDIEKKVNSRCEQIREAIKDTT
BAG39717.1        TEQLGIKLEKVNDTSKLGTANRVIVTKDHTTIVHDKNNSDIEKKVNSRCEQIREAIKDTT
M31887.1          TEQLGIKLEKVNDTSKLGTANRVIVTKDHTTIVHDKNNSDIEKKVNSRCEQIREAIKDTT
EF551292.1        TEQLGIKLEKVNDTSKLGTANRVIVTKDHTTIVHDKNNSDIEKKVNSRCEQ---------
EF551295.1        TEQLGIKLEKVNDTSKLGTANRVIVTKDHTTIVHDKNNSDIEKKVNSRCEQ---------
EF551296.1        TEQLGIKLEKVNDTSKLGTANRVIVTKDHTTIVHDKNNSDIEKKVNSRCEQ---------
EF551297.1        TEQLGIKLEKVNDTSKLGTANRVIVTKDHTTIVHDKNNSDIEKKVNSRCEQ---------
EF551298.1        TEQLGIKLEKVNDTSKLGTANRVIVTKDHTTIVHDKNNSDIEKKVNSRCEQ---------
EF551299.1        TEQLGIKLEKVNDTSKLGTANRVIVTKDHTTIVHDKNNSDIEKKVNSRCEQ---------
EF551301.1        TEQLGIKLEKVNDTSKLGTANRVIVTKDHTTIVHDKNNSDIEKKVNSRCEQ---------
EF551302.1        TEQLGIKLEKVNDTSKLGTANRVIVTKDHTTIVHDKNNSDIEKKVNSRCEQ---------
EF551304.1        TEQLGIKLEKVNDTSKLGTANRVIVTKDHTTIVHDKNNSDIEKKVNSRCEQ---------
EF551306.1        TEQLGIKLEKVNDTSKLGTANRVIVTKDHTTIVHDKNNSDIEKKVNSRCEQ---------
EF551307.1        TEQLGIKLEKVNDTSKLGTANRVIVTKDHTTIVHDKNNSDIEKKVNSRCEQ---------
EF551309.1        TEQLGIKLEKVNDTSKLGTANRVIVTKDHTTIVHDKNNSDIEKKVNSRCEQ---------
EF551290.1        TEQLGIKLEKVNDTSKLGTANRVIVTKDHTTIVHDKNNSDIEKKVNSRCEQ---------
EF551293.1        TEQLGIKLEKVNDTSKLGTANRVIVTKDHTTIVHDKNNSDIEKKVNSRCEQ---------
EF551294.1        TEQLGIKLEKVNDTSKLGTANRVIVTKDHTTIVHDKNNSDIEKKVNSRCEQ---------
EF551300.1        TEQLGIKLEKVNDTSKLGTANRVIVTKDHTTIVHDKNNSDIEKKVNSRCEQ---------
EF551305.1        TEQLGIKLEKVNDTSKLGTANRVIVTKDHTTIVHDKNNSDIEKKVNSRCEQ---------
EF551308.1        TEQLGIKLEKVNDTSKLGTANRVIVTKDHTTIVHDKNNSDIEKKVNSRCEQ---------
EF551289.1        TEQLGIKLEKVNDTSKLGTANRVIVTKDHTTIVHDKNNSDIEKKVNSRCEQ---------
EF551291.1        TEQLGIKLEKVNDTSKLGTANRVIVTKDHTTIVHDKNNSDIEKKVNSRCEQ---------
```

```
AY191585.1      TEQLGIKLEKVNDTSKLGTANRVIVTKDHTTIVHDKNNSDIEKKVNSRCEQIREAIKDTT
EF551288.1      TEQLGIKLEKVNDTSKLGTANRVIVTKDHTTIVHDKNNSDIEKKVNSRCEQ---------
AY059015.1      TEQLGIKLEKVNDTSKLGTANRVIVTKDHTTIVHDKNNSDIEKKVNSRCEQIREAIKDTT
AY191587.1      TEQLGIKLEKVNDTSKLGTANRVIVTKDHTTIVHDKNNSDIEKKVNSRCEQIREAIKDTT
                ***************************************************
                TEQLGIKLEKVNDTSKLGTANRVIVTKDHTTIVHDKNNSDIEKKVNSRCEQIREAIKDTT

AY191588.1      SDYEKEK-----------------------------------------------------
AY191589.1      SDYDKEK-----------------------------------------------------
EF551310.1      ------------------------------------------------------------
EF551303.1      ------------------------------------------------------------
AY191586.1      SDYEKEK-----------------------------------------------------
CAM79983.1      SDYEKEKLQERLAKLRNGVAVLKVGGATEVEQKERKDRVEDALHATRAAVEEGIVPGGGV
BAG39717.1      SDYEKEKLQERLAKLRNGVAVLKVGGATEVEQKERKDRVEDALHATRAAVEEGIVPGGGV
M31887.1        SDYEKEKLQERLAKLRNGVAVLKVGGATEVEQKERKDRVEDALHATRAAVEEGIVPGGGV
EF551292.1      ------------------------------------------------------------
EF551295.1      ------------------------------------------------------------
EF551296.1      ------------------------------------------------------------
EF551297.1      ------------------------------------------------------------
EF551298.1      ------------------------------------------------------------
EF551299.1      ------------------------------------------------------------
EF551301.1      ------------------------------------------------------------
EF551302.1      ------------------------------------------------------------
EF551304.1      ------------------------------------------------------------
EF551306.1      ------------------------------------------------------------
EF551307.1      ------------------------------------------------------------
EF551309.1      ------------------------------------------------------------
EF551290.1      ------------------------------------------------------------
EF551293.1      ------------------------------------------------------------
EF551294.1      ------------------------------------------------------------
EF551300.1      ------------------------------------------------------------
EF551305.1      ------------------------------------------------------------
EF551308.1      ------------------------------------------------------------
EF551289.1      ------------------------------------------------------------
EF551291.1      ------------------------------------------------------------
AY191585.1      SDYEKEK-----------------------------------------------------
EF551288.1      ------------------------------------------------------------
AY059015.1      SDYEKEK-----------------------------------------------------
AY191587.1      SDYEKEK-----------------------------------------------------

                SDYEKEKLQERLAKLRNGVAVLKVGGATEVEQKERKDRVEDALHATRAAVEEGIVPGGGV

AY191588.1      ------------------------------------------------------------
AY191589.1      ------------------------------------------------------------
EF551310.1      ------------------------------------------------------------
EF551303.1      ------------------------------------------------------------
AY191586.1      ------------------------------------------------------------
CAM79983.1      ALFYASRVLDSLKFDNEDQRVGINIIKKVLEAPVRQIVKNAGGKEDVVVNELSKSNDKNR
BAG39717.1      ALFYASRVLDSLKFDNEDQRVGINIIKKVLEAPVRQIVKNAGGKEDVVVNELSKSNDKNR
M31887.1        ALFYASRVLDSLKFDNEDQRVGINIIKKVLEAPVRQIVKNAGGKEDVVVNELSKSTDKNR
EF551292.1      ------------------------------------------------------------
EF551295.1      ------------------------------------------------------------
EF551296.1      ------------------------------------------------------------
EF551297.1      ------------------------------------------------------------
EF551298.1      ------------------------------------------------------------
EF551299.1      ------------------------------------------------------------
EF551301.1      ------------------------------------------------------------
EF551302.1      ------------------------------------------------------------
EF551304.1      ------------------------------------------------------------
EF551306.1      ------------------------------------------------------------
EF551307.1      ------------------------------------------------------------
EF551309.1      ------------------------------------------------------------
EF551290.1      ------------------------------------------------------------
EF551293.1      ------------------------------------------------------------
EF551294.1      ------------------------------------------------------------
EF551300.1      ------------------------------------------------------------
EF551305.1      ------------------------------------------------------------
EF551308.1      ------------------------------------------------------------
```

```
EF551289.1      ------------------------------------------------------------
EF551291.1      ------------------------------------------------------------
AY191585.1      ------------------------------------------------------------
EF551288.1      ------------------------------------------------------------
AY059015.1      ------------------------------------------------------------
AY191587.1      ------------------------------------------------------------

                ALFYASRVLDSLKFDNEDQRVGINIIKKVLEAPVRQIVKNAGGKEDVVVNELSKSTDKNR

AY191588.1      ------------------------------------------------------------
AY191589.1      ------------------------------------------------------------
EF551310.1      ------------------------------------------------------------
EF551303.1      ------------------------------------------------------------
AY191586.1      ------------------------------------------------------------
CAM79983.1      GFDARTMQYVDMIKAGIVDPTKVVRTALQDAFSVASLVIATSAMITDHEEDNNT-NRSGG
BAG39717.1      GFDARTMQYVDMIKAGIVDPTKVVRTALQDAFSVASLVIATSAMITDHEEDNNTGNRSGG
M31887.1        GFDARTMQYVDMIKAGIVDPTKVVRTALQDAFSVASLVIATSAMITDHEEDNNTGNRSGG
EF551292.1      ------------------------------------------------------------
EF551295.1      ------------------------------------------------------------
EF551296.1      ------------------------------------------------------------
EF551297.1      ------------------------------------------------------------
EF551298.1      ------------------------------------------------------------
EF551299.1      ------------------------------------------------------------
EF551301.1      ------------------------------------------------------------
EF551302.1      ------------------------------------------------------------
EF551304.1      ------------------------------------------------------------
EF551306.1      ------------------------------------------------------------
EF551307.1      ------------------------------------------------------------
EF551309.1      ------------------------------------------------------------
EF551290.1      ------------------------------------------------------------
EF551293.1      ------------------------------------------------------------
EF551294.1      ------------------------------------------------------------
EF551300.1      ------------------------------------------------------------
EF551305.1      ------------------------------------------------------------
EF551308.1      ------------------------------------------------------------
EF551289.1      ------------------------------------------------------------
EF551291.1      ------------------------------------------------------------
AY191585.1      ------------------------------------------------------------
EF551288.1      ------------------------------------------------------------
AY059015.1      ------------------------------------------------------------
AY191587.1      ------------------------------------------------------------

                GFDARTMQYVDMIKAGIVDPTKVVRTALQDAFSVASLVIATSAMITDHEEDNNTGNRSGG

AY191588.1      ---------------
AY191589.1      ---------------
EF551310.1      ---------------
EF551303.1      ---------------
AY191586.1      ---------------
CAM79983.1      GVGGGHHGGMGGMDF
BAG39717.1      GVGGGHHGGMGGMDF
M31887.1        GVGGGHHGGMGGMDF
EF551292.1      ---------------
EF551295.1      ---------------
EF551296.1      ---------------
EF551297.1      ---------------
EF551298.1      ---------------
EF551299.1      ---------------
EF551301.1      ---------------
EF551302.1      ---------------
EF551304.1      ---------------
EF551306.1      ---------------
EF551307.1      ---------------
EF551309.1      ---------------
EF551290.1      ---------------
EF551293.1      ---------------
EF551294.1      ---------------
EF551300.1      ---------------
```

```
EF551305.1      ----------------
EF551308.1      ----------------
EF551289.1      ----------------
EF551291.1      ----------------
AY191585.1      ----------------
EF551288.1      ----------------
AY059015.1      ----------------
AY191587.1      ----------------

                GVGGGHHGGMGGMDF
```

Consensus sequence of the GroEL chaperonin of *O. tsutsugamushi*

```
MSKQIVHSDQCRKKIIEGINVVANAVGITLGPKGRCVAIEQSYGPPKITKDGVSVAKAIQLKDKSLNVGAQFVISVASKT
ADVAGDGTTTATVIADAAVRELNKAEVAGIDIQEVRKGAEKAVEAVIADVRKNSSPVKNEEEIAQVATVSSNGDREIGEK
IANAMKQVGQEGVITVEDSKNFNFEVEVVKGMRFDRGYISQYFATNREKMITEFENPYILLLDQKVSTVQPLVPVLEAVA
HTGKPLVLIADDVDGEALTALILNNLKGSIKVVAVKAPGFGDRKKEMLEDIAILTNGEVITEQLGIKLEKVNDTSKLGTA
NRVIVTKDHTTIVHDKNNSDIEKKVNSRCEQIREAIKDTTSDYEKEKLQERLAKLRNGVAVLKVGGATEVEQKERKDRVE
DALHATRAAVEEGIVPGGGVALFYASRVLDSLKFDNEDQRVGINIIKKVLEAPVRQIVKNAGGKEDVVVNELSKSTDKNR
GFDARTMQYVDMIKAGIVDPTKVVRTALQDAFSVASLVIATSAMITDHEEDNNTGNRSGGGVGGGHHGGMGGMDF
```

Figure S1. CLUSTAL Omega (1.2.0) multiple sequence alignment of GroEL chaperonin of *O. tsutsugamushi* and the consensus sequence.

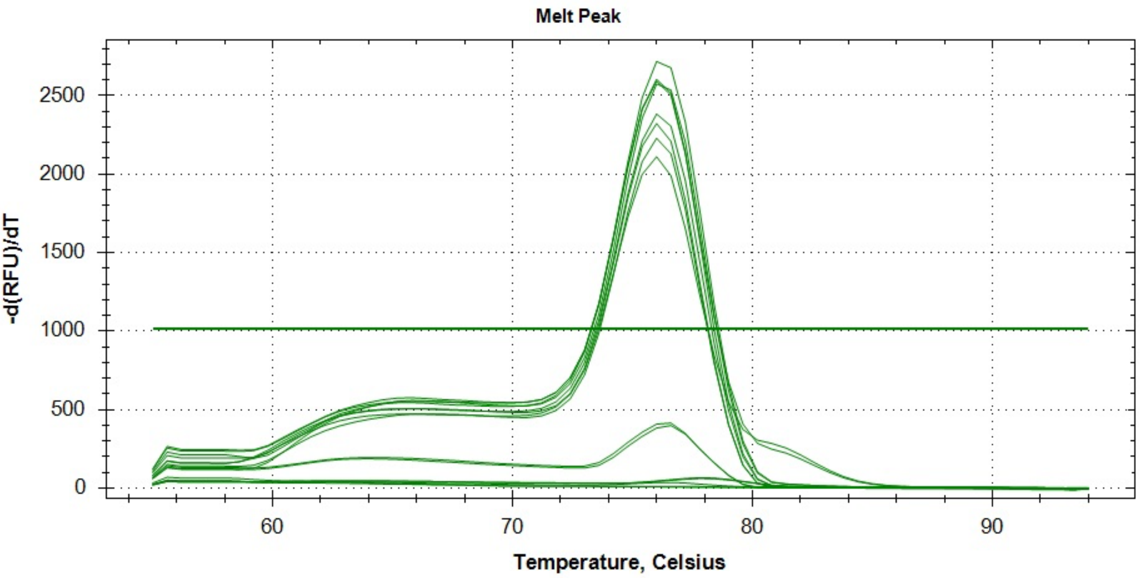

Figure S2. Melting curve of real-time PCR for detecting gene coding for *O. tsutsugamushi* GroEL. Melting curve analysis was performed with increments of 1 °C/step (72–95 °C) to determine the change in peak fluorescence over time (dF/dT).

Table S1. Performance of the ICT AgTK on the 40 frozen serum samples compared with the original diagnoses based on clinical features and laboratory tests including *Leptospira* IFA for leptospirosis and *O. tsutsugamushi* IFA for scrub typhus (Department of Medical Sciences, Ministry of Public Health, Thailand) and Dengue NS1 antigen and Dengue IgG/IgM ELISA for Dengue fever (SD Biosensor, Korea). ST = scrub typhus diagnosis; DF = Dengue fever diagnosis; LT = leptospirosis diagnosis.

No.	Code	Original diagnosis	*Leptospira* IFA		Dengue NS1 antigen	Dengue IgG/IgM ELISA		*O. tsutsugamushi* IFA		ICT AgTK
			**IgM**	**IgG**	**NS1**	**IgM**	**IgG**	**IgM**	**IgG**	**GroEL**
1	11-1-003	ST	<1:50	<1:50	Negative	<1:50	<1:50	1:3,200	1:100	Negative
2	11-1-004	ST	<1:50	<1:50	Negative	<1:50	<1:50	≤1:6400	≤1:6400	Positive
3	11-1-014	ST	<1:50	<1:50	Negative	<1:50	<1:50	≤1:6400	1:1600	Positive
4	11-1-035	ST	<1:50	<1:50	Negative	<1:50	<1:50	≤1:6400	1:800	Positive
5	11-1-118	ST	<1:50	<1:50	Negative	<1:50	<1:50	≤1:6400	≤1:6400	Positive
6	11-1-124	ST	<1:50	<1:50	Negative	<1:50	<1:50	≤1:6400	≤1:6400	Positive
7	11-1-154	ST	<1:50	<1:50	Negative	<1:50	<1:50	1:1600	≤1:6400	Positive
8	11-1-171	ST	<1:50	<1:50	Negative	<1:50	<1:50	1:800	1:3200	Positive
9	11-1-174	ST	<1:50	<1:50	Negative	<1:50	<1:50	1:800	1:1600	Positive
10	11-1-179	ST	<1:50	<1:50	Negative	<1:50	<1:50	1:3200	1:3200	Positive
11	11-1-192	ST	<1:50	<1:50	Negative	<1:50	<1:50	≤1:6400	1:800	Negative
12	11-1-227	ST	<1:50	<1:50	Negative	<1:50	<1:50	≤1:6400	1:3200	Positive
13	11-1-247	ST	<1:50	<1:50	Negative	<1:50	<1:50	1:200	1:3200	positive
14	11-1-248	ST	<1:50	<1:50	Negative	<1:50	<1:50	1:400	≤1:6400	Positive
15	11-1-259	ST	<1:50	<1:50	Negative	<1:50	<1:50	1:800	1:800	Positive
16	11-1-286	ST	<1:50	<1:50	Negative	<1:50	<1:50	1:400	1:3200	Positive
17	11-1-295	ST	<1:50	<1:50	Negative	<1:50	<1:50	1:100	1:800	Positive
18	11-1-334	ST	<1:50	<1:50	Negative	<1:50	<1:50	<1:50	1:1600	Negative
19	11-1-353	ST	<1:50	<1:50	Negative	<1:50	<1:50	1:1600	1:1600	Positive
20	11-1-379	ST	<1:50	<1:50	Negative	<1:50	<1:50	1:400	1:1600	Positive
21	4-0079-2	DF	<1:50	<1:50	Positive	<1:50	<1:50	<1:50	<1:50	Negative
22	4-0623	DF	<1:50	<1:50	positive	<1:50	<1:50	<1:50	<1:50	Negative
23	4-0640	DF	<1:50	<1:50	Positive	<1:50	<1:50	<1:50	<1:50	Negative
24	4-0651	DF	<1:50	<1:50	Positive	<1:50	<1:50	<1:50	<1:50	Negative
25	4-0660	DF	<1:50	<1:50	Positive	<1:50	<1:50	<1:50	<1:50	Negative
26	4-0661	DF	<1:50	<1:50	Positive	<1:50	<1:50	<1:50	<1:50	Negative
27	4-0682	DF	<1:50	<1:50	Positive	<1:50	<1:50	<1:50	<1:50	Negative
28	4-0700	DF	<1:50	<1:50	positive	<1:50	<1:50	<1:50	<1:50	Negative
29	4-0706	DF	<1:50	<1:50	Positive	<1:50	<1:50	<1:50	<1:50	Negative
30	4-0718	DF	<1:50	<1:50	Positive	<1:50	<1:50	<1:50	<1:50	Negative
31	11-1-116	LT	1:800	1:1600	Negative	<1:50	<1:50	<1:50	<1:50	Negative
32	11-1-144	LT	≤1:6400	1:100	Negative	<1:50	<1:50	<1:50	<1:50	Negative
33	11-1-183	LT	1:200	1:200	Negative	<1:50	<1:50	<1:50	<1:50	Negative
34	11-1-187	LT	1:400	<1:50	Negative	<1:50	<1:50	<1:50	<1:50	Negative

No.	Code	Original diagnosis	*Leptospira* IFA		Dengue NS1 antigen	Dengue IgM/IgG ELISA		*O. tsutsugamushi* IFA		ICT AgTK
			IgM	IgG	NS1	IgM	IgG	IgM	IgG	GroEL
35	11-1-253	LT	1:400	1:50	Negative	<1:50	<1:50	<1:50	<1:50	Negative
36	11-1-272	LT	1:400	1:100	Negative	<1:50	<1:50	<1:50	<1:50	Negative
37	3607	LT	1:100	1:400	Negative	<1:50	<1:50	<1:50	<1:50	Negative
38	3261	LT	1:800	1:3200	Negative	<1:50	<1:50	<1:50	<1:50	Negative
39	2692	LT	1:100	1:400	Negative	<1:50	<1:50	<1:50	<1:50	Negative
40	2454	LT	1:400	1:100	Negative	<1:50	<1:50	<1:50	<1:50	Negative

Table S2. Results of the GRoEL detection by using the ICT AgTK and real-time PCR performed blindly in our laboratory in comparison to the laboratory diagnoses performed at laboratory of Nan Hospital using test kits from SD Biosensor, Korea.

Code no.	Age)years(	Sex	Clinical and lab diagnosis at Nan hospital	Scrub typhus real-time PCR using either plasma or buffy coat samples	Scrub typhus ICT AgTK using either plasma or buffy coat samples	Results of ICT AgTK using plasma
						T C
1	47	F	Scrub typhus	+	+	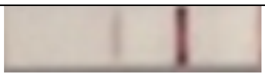
2	35	F	Dengue fever	-	-	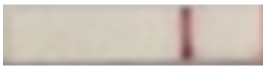
3	35	M	Leptospirosis	-	-	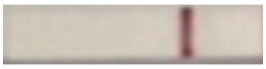
4	52	M	Scrub typhus	+	+	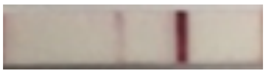
5	72	M	Leptospirosis	-	-	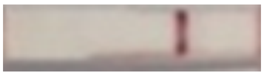
6	18	M	Dengue fever	-	-	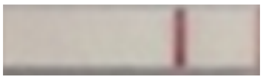
7	59	F	Dengue fever	-	-	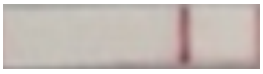
8	52	F	Scrub typhus	+	+	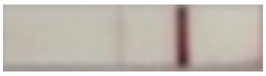
9	39	M	Scrub typhus	+	+	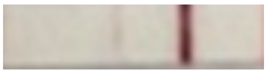
10	28	M	Scrub typhus	+	+	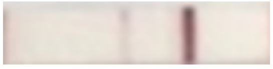
11	42	F	Scrub typhus	+	+	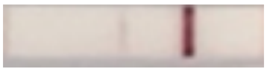
12	55	F	Scrub typhus	+	+	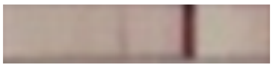
13	21	F	Scrub typhus	+	+	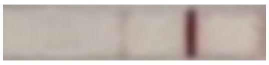
14	38	M	Scrub typhus	+	+	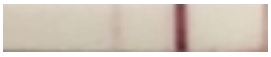
15	50	F	Scrub typhus	+	+	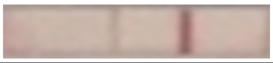

Table S3. Performance of the ICT AgTK evaluated independently at Prasat Hospital, Surin Province, by the hospital's medical technologist in comparison with the results of commercialized test kit (SD Biosensor, Korea) for detection of antibody (IgM/IgG) to *O. tsutsugamushi*.

**No.**	**IgM/IgG to *O. tsutsugamushi***	**ICT AgTK**	**ICT AgTK strip**<br>**T C**
1	IgM Positive<br>IgG Positive (weakly)	Positive	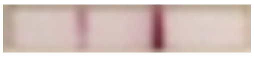
2	IgM Negative<br>IgG Negative	Negative	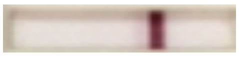
3	IgM Negative<br>IgG Negative	Negative	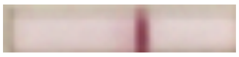
4	IgM Negative<br>IgG Negative	Negative	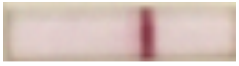
5	IgM Positive<br>IgG Negative	Positive	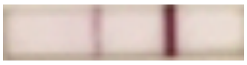
6	IgM Positive<br>IgG Negative	Weakly positive	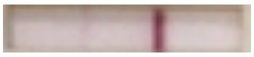
7	IgM Negative<br>IgG Positive	Positive	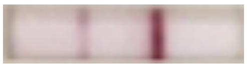
8	IgM Positive<br>IgG Negative	Positive	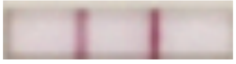
9	IgM Negative<br>IgG Negative	Negative	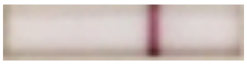
10	IgM Positive (weakly)<br>IgG Positive (weakly)	Positive	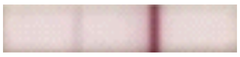
11	IgM Negative<br>IgG Negative	Negative	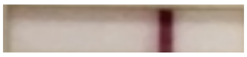
12	IgM Negative<br>IgG Negative	Negative	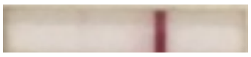
13	IgM Negative<br>IgG Negative	Negative	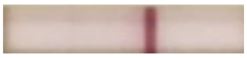
14	IgM Negative<br>IgG Negative	Negative	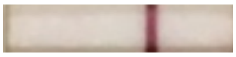
15	IgM Negative<br>IgG Negative	Negative	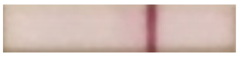
16	IgM Negative<br>IgG Negative	Negative	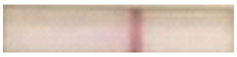
17	IgM Negative<br>IgG Negative	Negative	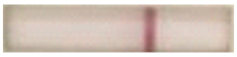
18	IgM Negative<br>IgG Negative	Negative	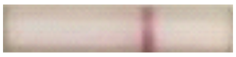
19	IgM Negative<br>IgG Negative	Negative	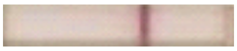
20	IgM Negative<br>IgG Negative	Negative	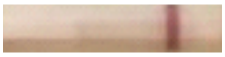
